# Epileptic spasms relapse is associated with response latency but not conventional attributes of post‐treatment EEG

**DOI:** 10.1002/epi4.12931

**Published:** 2024-04-08

**Authors:** Emmi Deckard, Rujuta Sathe, David Tabibzadeh, Aria Terango, Aran Groves, Rajsekar R. Rajaraman, Hiroki Nariai, Shaun A. Hussain

**Affiliations:** ^1^ Department of Pediatrics Division of Neurology University of California Los Angeles and UCLA Mattel Children's Hospital California Los Angeles USA

**Keywords:** epileptiform discharges, infantile spasms, vigabatrin, west syndrome

## Abstract

**Objective:**

Relapse of epileptic spasms after initial treatment of infantile epileptic spasms syndrome (IESS) is common. However, past studies of small cohorts have inconsistently linked relapse risk to etiology, treatment modality, and EEG features upon response. Using a large single‐center IESS cohort, we set out to quantify the risk of epileptic spasms relapse and identify specific risk factors.

**Methods:**

We identified all children with epileptic spasms at our center using a clinical EEG database. Using the electronic medical record, we confirmed IESS syndrome classification and ascertained treatment, response, time to relapse, etiology, EEG features, and other demographic factors. Relapse‐free survival analysis was carried out using Cox proportional hazards regression.

**Results:**

Among 599 children with IESS, 197 specifically responded to hormonal therapy and/or vigabatrin (as opposed to surgery or other second‐line treatments). In this study, 41 (21%) subjects exhibited relapse of epileptic spasms within 12 months of response. Longer duration of IESS prior to response (>3 months) was strongly associated with shorter latency to relapse (hazard ratio = 3.11; 95% CI 1.59–6.10; *p* = 0.001). Relapse was not associated with etiology, developmental status, or any post‐treatment EEG feature.

**Significance:**

This study suggests that long duration of IESS before response is the single largest clinical predictor of relapse risk, and therefore underscores the importance of prompt and successful initial treatment. Further study is needed to evaluate candidate biomarkers of epileptic spasms relapse and identify treatments to mitigate this risk.

**Plain Language Summary:**

Relapse of infantile spasms is common after initially successful treatment. With study of a large group of children with infantile spasms, we determined that relapse is linked to long duration of infantile spasms. In contrast, relapse was not associated with the cause of infantile spasms, developmental measures, or EEG features at the time of initial response. Further study is needed to identify tools to predict impending relapse of infantile spasms.

**Key points**

- Time to epileptic spasms relapse was shorter among patients with longer duration of infantile epileptic spasms syndrome (IESS).
- Post‐treatment EEG features were not associated with time to epileptic spasms relapse.
- This is the largest study of children with IESS that specifically addresses relapse.

## 1 INTRODUCTION

Infantile epileptic spasms syndrome (IESS) is a potentially devastating form of developmental and epileptic encephalopathy and the most common epilepsy syndrome in the first year of life.^1^ IESS typically manifests with epileptic spasms, hypsarrhythmia (including variations thereof), and neurodevelopmental arrest or regression.^2^ Long‐term outcomes are often poor, with high rates of refractory epilepsy (i.e., Lennox–Gastaut syndrome),^3^ intellectual disability,^4^ and autism.^5^ Poor long‐term developmental outcomes are multifactorial in origin and have been specifically associated with etiology,^4^ latency to initial treatment,^6^ and lack of sustained remission of epileptic spasms.^7^ Despite favorable short‐term response to hormonal therapy (corticosteroids and adrenocorticotropic hormone) and vigabatrin,^8^ epileptic spasms relapse is common and often occurs upon discontinuation of initial treatment.^9^ Relatively early discontinuation of effective therapy is chiefly motivated by concern for adverse consequences of long‐term use; hormonal therapy confers numerous risks (especially immunosuppression and sepsis),^10^ and vigabatrin has been linked to irreversible peripheral vision loss^11^ as well as adverse MRI phenomena.^12^, ^13^

As reviewed in the 2004 Infantile Spasms Treatment Practice Parameter, the cumulative risk of relapse of ES following treatment with ACTH (14 studies), corticosteroids (5 studies), and vigabatrin (14 studies) is substantial (approximately 30%), with marked variation as a function of follow‐up duration (higher rates with longer follow‐up) and etiology (higher rates with *symptomatic* than *cryptogenic* classification).^9^ Of note, we refer to *symptomatic* and *cryptogenic* etiology in this report to facilitate comparison to prior studies, acknowledging that these terms are problematic in contemporary use.^14^ Upon achieving response with initial therapy, several small studies suggest that relapse may be associated with post‐response EEG features^15^ and—at least in specific etiologic subgroups—relapse risk may be modified by extended treatment with hormonal therapy^7^ or vigabatrin.^16^ In this study, we set out to evaluate risk factors for epileptic spasms relapse in a large, single‐center cohort.

## 2 METHODS

### 2.1 Institutional approvals

This study was approved by the institutional review board at the University of California, Los Angeles.

### 2.2 Hypotheses

We hypothesized that relapse would be more frequent among children with (1) known etiology or abnormal development prior to IESS onset (i.e., lack of *cryptogenic* etiology), (2) long duration of epileptic spasms prior to treatment response, and (3) the presence of frequent, multifocal epileptiform discharges on post‐treatment EEG.

### 2.3 Subject selection and data acquisition

All data were abstracted from the electronic medical record. Using a clinical EEG database, we identified all patients who have been evaluated for IESS at UCLA Mattel Children's Hospital between 2001 and 2022. For each subject, systematic chart review was conducted to code response to treatment (defined as freedom from epileptic spasms and hypsarrhythmia for at least 1 month, beginning not more than 1 month after initiation of a specific treatment regimen) and latency to relapse (if any). In addition to demographic and etiologic variables, we specifically queried treatment modality at response (corticosteroids, ACTH, vigabatrin, or other), and cataloged electrographic features from the post‐treatment EEG (i.e., presence and topographic distribution of epileptiform discharges and slowing). For those cases in which EEG reports were available and sufficiently detailed, we quantified the burden of sporadic epileptiform discharges using the American Clinical Neurophysiology Society rubric as rare (<1/h), occasional (>1/h and <1/min), frequent (>1/min and <1/page), and abundant (>1/page).^17^ To be included in the analysis, the EEG establishing response had to be conducted within 1 month of treatment initiation.

### 2.4 Statistical methods

Continuous summary data were presented as median and interquartile range (IQR) based on nonparametric distributions. Comparisons of continuous and dichotomous variables were accomplished with the Wilcoxon rank‐sum test and the Fisher exact test, respectively. Survival analysis evaluating time to epileptic spasms relapse was accomplished with the Kaplan–Meier procedure and Cox proportional hazards regression. Given the age‐specificity of epileptic spasms relapse and observation that the proportional hazards assumption was violated beyond 12 months of follow‐up, all follow‐up data were censored at 12 months following response. Given that age at response and duration of IESS exhibited skewed distributions, these continuous variables were dichotomized using an approximately median split when evaluated with Cox proportional hazards regression. All comparisons were two‐sided and only *p‐*values less than 0.05 were considered statistically significant. Statistical calculations were facilitated with STATA software (Statacorp, version 14, College Station, Texas, USA).

## 3 RESULTS

### 3.1 Subjects

We identified 677 children with epileptic spasms, of whom 599 exhibited IESS based on age of onset (1–24 months) and absence of alternative epilepsy syndrome classification (e.g., Ohtahara syndrome). Overall, 321 patients exhibited response to any therapy observed at UCLA, including 43 with previous treatment and response (not confirmed by the study team) at other centers before the first UCLA encounter. Only 208 patients had EEG‐confirmed epileptic spasms and responded to “standard therapy” (i.e., prednisolone, ACTH, and/or vigabatrin) at our center; the remainder exhibited resolution of epileptic spasms with surgical resection or second‐line medications. Eleven subjects were excluded because of incomplete clinical data in the medical record. As such, 197 subjects were included in this study. Clinical and demographic characteristics of the study population are summarized in Table 1. Of note, patients who went on to relapse were older and exhibited a longer duration of epileptic spasms. Detailed EEG data (i.e., sufficient to classify abundance and topographic distribution of post‐treatment epileptiform discharges) was available for only 109 subjects; among the excluded cases, a qualitative description of EEG results indicated absence of both epileptic spasms and hypsarrhythmia, but lacked further quantitative detail. The burden and distribution of interictal slowing is illustrated in Table 2. With respect to these attributes, there were no significant differences between patients with and without relapse.

**TABLE 1:** Characteristics of the study population.

	No relapse (*n* = 156)	Relapse (*n* = 41)	*p*‐Value
Demographics and epilepsy characteristics			
Female, *n* (%)	72 (46%)	13 (32%)	0.11
Age of onset of infantile spasms, months^a^	6.1 (4.0, 8.9)	7.0 (4.4, 11.1)	0.48
Duration of infantile spasms before initial response, months^a^	1.9 (0.7, 6.6)	4.4 (2.1, 9.0)	0.007
Age at initial response, months^a^	9.5 (6.7, 16.4)	13.6 (9.2, 19.5)	0.01
Prior response at other centers^b^	15 (10%)	3 (7%)	1.00
Time to relapse, months^a^	—	3.4 (1.9, 5.8)	—
Development			
Normal development at onset of epileptic spasms, *n* (%)	66 (42%)	19 (46%)	0.72
Etiology			
Known etiology	102 (65%)	29 (71%)	0.58
Structural, *n* (%)	74 (47.7%)	21 (51.2%)	0.73
Genetic, *n* (%)	48 (31%)	11 (27%)	0.70
Unknown etiology and normal development before IESS (formerly *cryptogenic*), *n* (%)	25 (16%)	5 (12%)	0.63
Treatment at initial response			
Hormonal therapy alone, *n* (%)	74 (47%)	27 (66%)	0.05
Vigabatrin alone, *n* (%)	51 (33%)	9 (22%)	0.25
Combination therapy (hormonal therapy and vigabatrin, *n* (%)	31 (20%)	5 (12%)	0.36
Post‐treatment course			
Total follow‐up, months^a^	48.1 (14.5, 90.6)	59.6 (38.6, 113.1)	0.05

^a^ Median (interquartile range).

^b^ These patients had reported (but unconfirmed) response to treatment at other centers prior to first UCLA encounter, with subsequent EEG‐confirmed relapse and response at UCLA.

**TABLE 2:** EEG features at response.

	No relapse (*n* = 89)	Relapse (*n* = 20)	*p*‐Value
EEG demonstrating response available for review, *n* (%)	89 (57%)	20 (49%)	0.38
Epileptiform discharges present, *n* (%)	61 (69%)	16 (80%)	0.42
At least “frequent” epileptiform discharges, *n* (%)	34 (38%)	11 (55%)	0.21
Multifocal epileptiform discharges, *n* (%)	37 (42%)	10 (50%)	0.62
Generalized epileptiform discharges, *n* (%)	11 (12%)	3 (15%)	0.72
Multifocal or generalized discharges, *n* (%)	39 (44%)	10 (50%)	0.62
Frequent, multifocal, or generalized discharges, *n* (%)	27 (30%)	8 (40%)	0.43
Slowing present, *n* (%)	45 (51%)	10 (50%)	1.00
Generalized slowing, *n* (%)	25 (28%)	7 (35%)	0.59
Focal slowing, *n* (%)	20 (22%)	3 (15%)	0.56

### 3.2 Relapse‐free survival analyses

**FIGURE 1:**
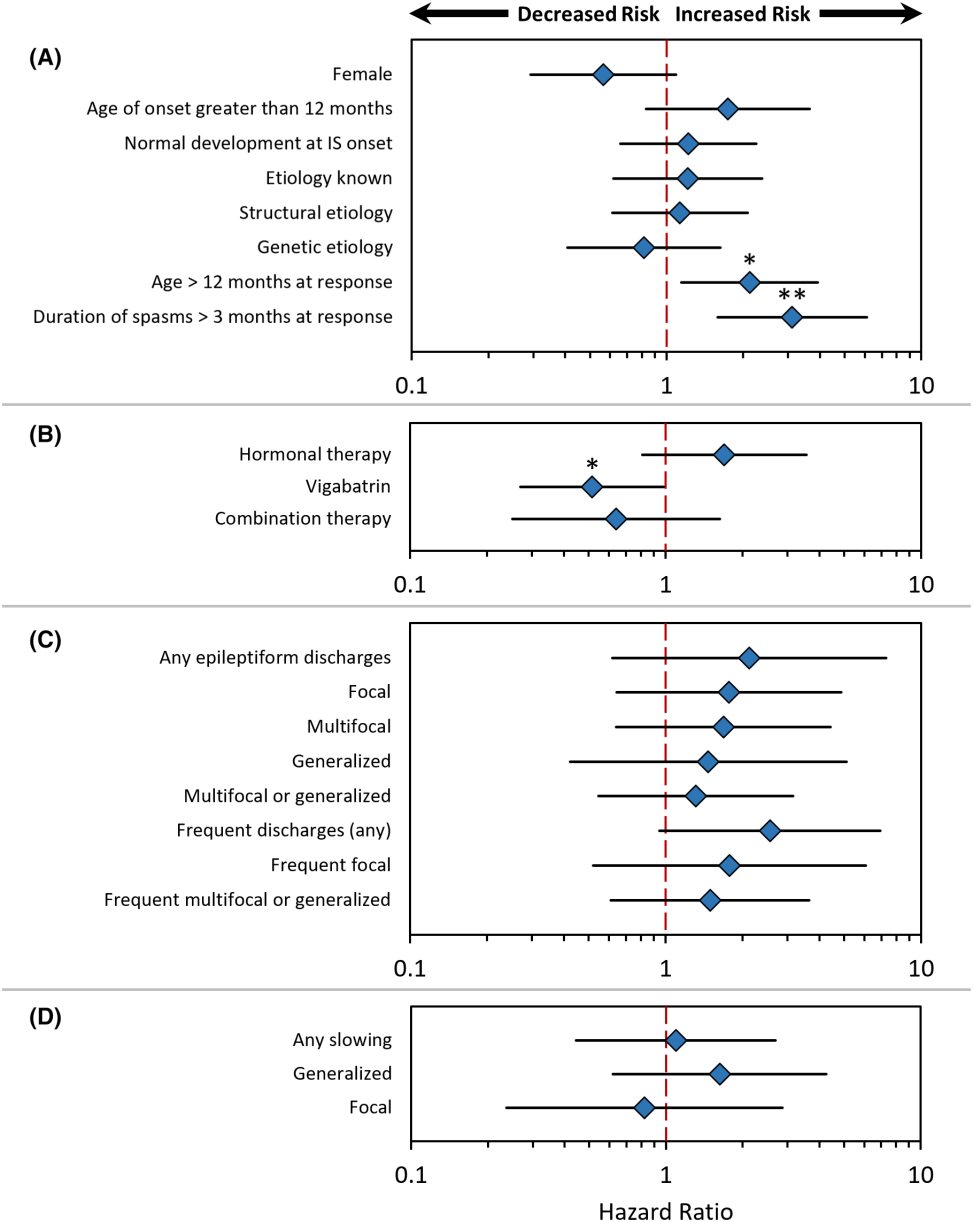
Clinical and post‐treatment EEG associations with relapse. Modified forest plots illustrate sequential univariate evaluation, via Cox proportional hazards regression, of the association between latency to relapse and demographic/clinical parameters (A), treatment attributes at response (B), burden of epileptiform discharges on post‐treatment EEG (C), and burden of interictal slowing on post‐treatment EEG (D). Each diamond represents the point estimate of the hazard ratio, and error bars represent the 95% confidence interval. *p < 0.05; **p < 0.01.

In this study, 41 (21%) subjects exhibited relapse of epileptic spasms within 12 months of response. The results of relapse‐free survival analysis are summarized in Figure 1. Only one of our a priori hypotheses was upheld. Longer duration of IESS (>3 months at the time of response) was associated with a threefold increased hazard of relapse (HR 3.11; 95% CI 1.59–6.10; *p* = 0.001). With regard to etiology, latency to epileptic spasms relapse was not associated with known etiology, abnormal development at IESS onset, or both in combination. Similarly, the presence of frequent multifocal or generalized discharges was not associated with latency to relapse.

On an exploratory basis, and as illustrated in Figure 1A, we screened several demographic variables for association with latency to relapse. Likely mirroring the aforementioned association between relapse and IESS duration, we observed that subjects who were older than 12 months at the time of response exhibited elevated hazard of relapse (HR 2.12; 95% CI 1.14–3.93; *p* = 0.017). Indeed, longer duration of IESS and age at response were highly correlated (spearman rho 0.43, *p* < 0.0001). However, history of prior response at other centers (followed by relapse and UCLA‐confirmed response, *n* = 18) was not associated with latency to relapse (HR 0.76; 95% CI 0.23–2.47, *p* = 0.65). In addition, we observed a trend toward lower risk of relapse among females (HR = 0.56; 95% CI 0.29–1.09; *p* = 0.09). Regarding treatment, as demonstrated in Figure 1B, we observed an association such that responders to vigabatrin (defined as patients whose regimen at time of response included vigabatrin, monotherapy or otherwise) were less likely to experience relapse (HR = 0.52, 95% CI 0.27–0.99; *p* = 0.045). In evaluating post‐treatment EEG attributes, there were no associations between latency to relapse and the presence, distribution, or abundance of epileptiform discharges (Figure 1C) or the presence and distribution of interictal slowing (Figure 1D). Of note, although not statistically significant, the point estimate of the hazard ratio for all measures of epileptiform discharge burden were greater than 1.0, and 95% confidence intervals tended to be large.

## 4 DISCUSSION

To our knowledge, this is the largest study to specifically evaluate potential risk factors for epileptic spasms relapse. Although we have reported a substantial risk of relapse, and identified several risk factors for relapse, this study is most noteworthy for the relative lack of identified risk factors. Foremost, we did not replicate prior studies linking relapse to etiology, developmental status at IESS onset, or any interictal EEG attribute on post‐treatment EEG. Our observation that relapse is associated with duration of IESS supports the concept that prompt diagnosis and treatment of IESS is essential. The paucity of other identified risk factors highlights the significant unmet needs for (1) biomarkers to identify relapse risk, and (2) novel treatment approaches to prevent relapse.

It is important to note that this study is methodologically limited on several fronts. Foremost, the study was retrospective, treatments before and after response were not randomized, and the sample size was relatively small within the context of the Cox proportional hazards regression—especially the analysis of the subgroup with adequate quantification of epileptiform discharge burden on post‐treatment EEG. With regard to our EEG analysis, it is possible that the subset of patients with adequate EEG are systematically different (in an unmeasured fashion) from the broader cohort, and our analytic approach to EEG variables may thus introduce a selection bias. Further, our IESS cohort is not population based and it is unlikely to be representative of the broader IESS population. Given that the study was conducted at a tertiary referral center, our overall estimate of relapse risk may be high. Finally, although we focused on relapse—an important medium‐to long‐term outcome in the clinical management of IESS—we have ignored more important longer term outcomes including developmental and behavioral outcomes, emergence of other seizure‐types, and evolution to other epilepsy syndromes (i.e., Lennox–Gastaut Syndrome).

Although we have linked epileptic spasms risk to duration of IESS before response, the association lacks mechanistic precision. Given that many patients began treatment at other centers, and details of these treatments were not consistently recapitulated in our center's medical record, we were often unable to determine if initial treatments were “standard” (i.e., utilized appropriate dosage and duration of hormonal therapy or vigabatrin) or explicitly calculate latency from IESS onset to initiation of these treatments. As a consequence, our quantification of IESS duration includes latency from IESS onset to diagnosis, latency from diagnosis to first standard therapy, and in some cases, latency to subsequent medication trials intermixed with intervals of suspected clinical (though not necessarily electrographic) remission. Thus, although we have determined that latency from onset to confirmed response is associated with release, we do not know the relative impact of the constituent subintervals.

Our lack of observed association with etiology is not necessarily surprising, especially given the evolution of genetic diagnostics over the span of this study. In addition, whereas some etiologies may increase risk of relapse, relapse itself likely motivates the conduct of genetic testing and utilization of those diagnostic platforms with highest yield. Similarly, advances in neuroimaging have progressively facilitated identification of rather subtle structural abnormalities (e.g., ILAE type 1 focal cortical dysplasia), and these subtle abnormalities are likely detected most often among patients with treatment non‐response or relapse. Altogether, we suspect that some of our patients with relatively low risk of relapse would have been classified as *cryptogenic* in years past, but are now classified as structural or genetic as a result of contemporary diagnostic methods. This evolution in etiologic discovery would be expected to diminish the protective association between relapse and the outdated concept of *cryptogenic* etiology. Nevertheless, the potential links between specific etiologies and relapse warrant further study, ideally in a cohort in which etiologic evaluation does not vary as a function of treatment refractoriness.

With respect to a link between post‐response EEG and relapse, our lack of association may be related not only to the size of the cohort, but the timing of EEG acquisition as well. Our center's treatment protocol dictates EEG administration 2 weeks after the start of hormonal therapy or vigabatrin. We suspect EEG at this early timepoint often remains epileptiform—albeit not hypsarrhythmic—and continues to improve over subsequent weeks among sustained responders. Indeed, among the patients without relapse in our EEG analysis, 69% exhibited epileptiform discharges, and 38% exhibited frequent epileptiform discharges. Similarly, in the study of Hayashi and colleagues, more than half of patients exhibited continued epileptiform discharges on EEG in the 0–2 week range following successful ACTH therapy; most of these patients then displayed diminished topographic distribution and/or complete resolution of epileptiform discharges in the 1–3 month period following treatment.^15^

Our observation that response to vigabatrin was associated with lower risk of relapse should be interpreted with caution. Treatment selection was not randomized, and was often sequential (e.g., vigabatrin started after failure of hormonal therapy). Furthermore, we are not suggesting that vigabatrin is superior to hormonal therapy or combination therapy, as might be the impression from an isolated review of Figure 1B. To be clear, our group generally favors first‐line combination therapy based on the results of the International Collaborative Infantile Spasms Study (ICISS).^18^ To the extent that we observed an effect (i.e., latency to relapse was longer among patients whose response regimen included vigabatrin), this may reflect “milder” IESS disease burden. For example, patients who respond to vigabatrin might have less severe disease (and thus lower risk of relapse) than patients who require more effective initial therapy (i.e., hormonal therapy). In addition, our observation may indicate a benefit of continued vigabatrin after response. Given our impression that the risk of clinically significant vigabatrin‐associated visual field loss is low^19^ and that risk of the vigabatrin‐associated brain abnormalities on MRI are not associated with duration of treatment,^20^ we have devised a protocol in which patients continue vigabatrin for 12 months following response to any regimen that includes vigabatrin.^21^ However, although our observation suggests that continued vigabatrin may reduce risk of relapse, there are other conflicting data to consider. In line with our observation, relapse risk among a multicenter cohort of children with TSC (an etiologic subgroup with exceptional response to vigabatrin in general) was lower among patients with higher post‐response vigabatrin dosage.^16^ On the other hand, we did not observe a similar effect in an exploratory analysis (published as abstract only) of children without TSC,^22^ and relapse in ICISS (contrasting hormonal therapy alone versus the combination of hormonal therapy and vigabatrin) did not seem to vary as a function of post‐response vigabatrin exposure in an unpublished preliminary analysis.^23^ Additional study on the potential efficacy of post‐response vigabatrin is needed, especially given the adverse event profile of vigabatrin. Beyond vigabatrin, it is possible that long‐term, low‐dose hormonal therapy may be a superior epileptic spasms relapse prevention strategy. As this is not an approach employed at our center, we had no opportunity to evaluate it in this study. Nevertheless, the potential utility of long‐term hormonal therapy is suggested by the exceptionally favorable long‐term outcomes (normal IQ, lack of relapse) among a cohort of 22 children with IESS, with unknown etiology and normal development prior to the onset of IESS, who received prompt treatment with ACTH, followed by at least 6 months of low‐dose prednisolone.^7^

Given that adverse long‐term epilepsy and neurodevelopmental outcomes are common with IESS—even among the subgroup with initial response to treatment—it is critical that we develop therapeutic strategies to prevent these outcomes and identify novel biomarkers to facilitate targeted treatment of children at high risk. Potential biomarkers include conventional EEG abnormalities (e.g., increase in abundance or topographic distribution of epileptiform discharges^15^), and an array computational EEG features such as spectral power,^24^ long‐range temporal correlations,^25^ functional connectivity,^26^ coherence,^24^ entropy,^27^ and phase‐amplitude coupling.^28^ Until such tools and technology are developed and validated, the management of IESS after initial response to therapy will continue in a haphazard fashion, with little guidance for treatment and EEG surveillance.

## AUTHOR CONTRIBUTIONS

ED, HN, RRR, and SAH contributed to conceptualization. ED, RS, DT, AT, AG, and SAH contributed to data collection and curation. ED and SAH contributed to data analysis and drafting the manuscript. All authors participated in data interpretation and revision of the manuscript.

## CONFLICT OF INTEREST STATEMENT

Dr. Rajaraman has received research support from Marinus and the International CDKL5 Research Foundation. Dr. Nariai is supported by the National Institute of Neurological Disorders and Stroke (NINDS) K23NS128318, the Sudha Neelakantan & Venky Harinarayan Charitable Fund, the Elsie and Isaac Fogelman Endowment, and the UCLA Children's Discovery and Innovation Institute (CDI) Junior Faculty Career Development Grant (#CDI‐TTCF‐07012021). Dr. Hussain has received research support from the John C. Hench Foundation, the CJDA Foundation, the Mohammed F. Alibrahim Endowment, the Elsie and Isaac Fogelman Endowment, the Epilepsy Therapy Project, the Milken Family Foundation, Paul Hughes Family Foundation, the Pediatric Epilepsy Research Foundation, Eisai, Bio‐Pharm, Lundbeck, Insys, GW Pharmaceuticals, UCB Biopharma, Zogenix, Marinus, and the NIH. He has received compensation for service as a consultant to Amzell, Aquestive Therapeutics, Equilibre Biopharmaceuticals, Insys, GW Pharmaceuticals, Mallinckrodt, Marinus, MGC Pharmaceuticals, Radius, Shennox, UCB Biopharma, Upsher‐Smith Laboratories, West Therapeutic Development, and Zogenix. The remaining authors report no conflicts of interest. We confirm that we have read the Journal's position on issues involved in ethical publication and affirm that this report is consistent with those guidelines.
